# Ranking with submodular functions on a budget

**DOI:** 10.1007/s10618-022-00833-4

**Published:** 2022-04-23

**Authors:** Guangyi Zhang, Nikolaj Tatti, Aristides Gionis

**Affiliations:** 1 KTH Royal Institute of Technology, Stockholm, Sweden; 2 HIIT, University of Helsinki, Helsinki, Finland

**Keywords:** Ranking, Submodular maximization, Dynamic programming, Approximation algorithms

## Abstract

Submodular maximization has been the backbone of many important machine-learning problems, and has applications to viral marketing, diversification, sensor placement, and more. However, the study of maximizing submodular functions has mainly been restricted in the context of selecting a set of items. On the other hand, many real-world applications require a solution that is a ranking over a set of items. The problem of ranking in the context of submodular function maximization has been considered before, but to a much lesser extent than item-selection formulations. In this paper, we explore a novel formulation for ranking items with submodular valuations and budget constraints. We refer to this problem as *max-submodular ranking* ($\text {MSR}$). In more detail, given a set of items and a set of non-decreasing submodular functions, where each function is associated with a budget, we aim to find a ranking of the set of items that maximizes the sum of values achieved by all functions under the budget constraints. For the $\text {MSR}$ problem with cardinality- and knapsack-type budget constraints we propose practical algorithms with approximation guarantees. In addition, we perform an empirical evaluation, which demonstrates the superior performance of the proposed algorithms against strong baselines.

## Introduction

Combinatorial optimization plays a central role in many machine-learning problems. One prevalent approach to solve such problems is via *submodular-optimization* techniques. The popularity of submodular-optimization methods results from the fact that in many real-world settings the objective function exhibits the “diminishing returns” property, as well as from the ever-growing rich toolkit that has been developed in the past decades. One fundamental primitive in this toolkit is *submodular maximization* (Krause and Golovin 2014), which has been the backbone of a number of important problems, such as sensor placement (Krause et al. 2008), viral marketing in social networks (Kempe et al. 2015), document summarization (Lin and Bilmes 2011), and more.

Submodular optimization has mainly been studied in the context of *subset-selection problems*. However, in many real-world applications the goal is to find a *ranking* over a set of items. Finding a ranking is a significantly more challenging task than subset selection, as the search space is factorially larger. One successful attempt of applying ideas from submodular optimization to ranking is the *submodular-ranking* problem ($\text {SR}$) (Azar and Gamzu 2011). In this problem, given a set of items and a set of submodular functions, the goal is to find a (partial) ranking of the items so as to minimize the average “cover time” of all functions.

An exemplary application of $\text {SR}$ is in the *multiple intents re-ranking* problem (Azar et al. 2009), which has applications in web searching. In this problem setting, a user query may correspond to multiple user intents. For example, a query of “java” may mean a programming language, an island, or a type of coffee. Even for a seemingly unambiguous query, such as “New York,” there exist many possible intents, for example, attractions, cuisine, travel, cultural events, etc. In the absence of an explicit user intent, we need to consider all possibilities. The $\text {SR}$ formulation proposes to model each intent as a submodular function, whose value improves when a non-redundant web page of the right intent is encountered, and reaches a maximum when the user is satisfied, i.e., having gathered sufficient information. The goal is to produce a ranking of web pages that minimizes the expected number of pages a user has to browse before they satisfy their information needs. The expectation here is over the distribution of different user intents, which for this particular application can be assumed to be known.

While the $\text {SR}$ formulation can be useful in some cases, it fails to model realistically a number of other applications. Critically, it assumes that a demand can wait indefinitely before it gets satisfied. In the previous example, for instance, it is assumed that users will keep reading down a ranked list of web pages until they gather enough information. In reality, a budget can be set for the amount of service that a user receives. The budget can be the number of web pages to browse, or the time to spend on the web-search task. A user stops receiving service once the budget is exceeded. Moreover, the budget can vary across different demands. For example, a user intent can be classified into one of three types, informational, navigational, and transactional (Jansen et al. 2008), and each may come with a different budget, translating to the amount of “patience” that a user exhibit to obtain results for each type. User intents and budgets can be readily extracted from the past search logs.

To accommodate budgeted versions of the submodular ranking problem, we propose a new formulation, which we call *max-submodular ranking* ($\text {MSR}$). In the $\text {MSR}$ problem, we are given a set of non-decreasing submodular functions, each associated with a budget. We aim to find a ranking that, instead of minimizing the total coverage time of the functions, maximizes the sum of function values (coverage) under individual budget constraints. In other words, every item in the ranking incurs a cost, and each function is evaluated at the maximal prefix of the ranked sequence that does not exceed its budget. A precise formulation of the $\text {MSR}$ problem is provided in Sect. 3.

In this paper, we propose practical algorithms with approximation guarantees for $\text {MSR}$, when the budget constraints are either cardinality or knapsack constraints. We also note that the well-known *constrained submodular maximization* and *minimum submodular cover* problems are special cases of $\text {MSR}$ and $\text {SR}$, respectively, when there is a single submodular function. In this sense, the $\text {MSR}$ problem we define is a dual problem of $\text {SR}$, in the same way that *max* *k-cover* is a dual problem of *minimum set cover*.

$\text {MSR}$ has great potential to be applied in other scenarios, such as in the case where the submodular functions are 0–1 activation functions. We call this special case *max-activation ranking* ($\text {MAR}$) problem. The idea is to activate as many demands as possible with a common ranking of items, or services, under individual budget constraints. As an example, some subscription-based streaming media services, such as Netflix, produce content in a data-driven fashion. One possibility is to arrange the plot structure in a TV series such that the maximum number of audience will get interested before their individual cut-off points for a new show. The goal for the TV series producer is to encourage the maximum-size audience to continue watching. A plot structure can be characterized as a sequence of scenes, each described by a set of tags, such as romantic, adventurous, funny, etc., which may interest particular audience. Similar applications can also be found in ranking commercial ads, ranking customer reviews, creating play lists for music streaming services, and more.

In concrete, our contributions in this paper are summarized as follows.

- We introduce the novel problem of *max-submodular ranking* ($\text {MSR}$), where the goal is to find a ranking of a set of items so as to maximize the total value of a set of submodular functions under budget constraints.
- We prove that a simple greedy algorithm achieves a factor-2 approximation for the $\text {MSR}$ problem under cardinality constraints, which is tight for this particular greedy algorithm.
- We show that a weighted greedy algorithm that pays more attention to functions with small budget achieves a factor-3 approximation for the $\text {MSR}$ problem under cardinality constraints. While its worst-case bound is worse, there are natural problem instances for which the weighted greedy finds better solutions than its unweighted counterpart.
- We devise a new algorithm that returns the best solution among the solutions found by a cost-efficient greedy algorithm and a ranking of “large” items produced by dynamic programming. Our algorithm achieves an approximation factor arbitrarily close to 4 for the $\text {MSR}$ problem under knapsack constraints.
- We empirically evaluate and compare different algorithms on real-life datasets, and find that the proposed algorithms achieve superior performance when compared with strong baselines.

The rest of the paper is organized as follows. We start by discussing the related work in Sect. 2, and we formally introduce the $\text {MSR}$ problem in Sect. 3. The unweighted and weighted greedy algorithms for $\text {MSR}$ under cardinality constraints are presented and analyzed in Sects. 4.1 and 4.2, respectively. The novel algorithm for the $\text {MSR}$ problem under knapsack constraints is introduced and analyzed in Sect. 5. We present our empirical evaluation in Sect. 6, and we offer our concluding remarks in Sect. 7.

## Related work

### Submodular maximization

Submodular maximization is a special case of our formulation when given only a single function. Coupled with a non-decreasing property and with a cardinality constraint it is well-known that a simple greedy algorithm achieves a $e/(e-1)$ approximation (Nemhauser et al. 1978), which is also shown to be tight (Nemhauser and Wolsey 1978). For a more general budget constraint, a natural algorithm is to return the best solution among the solutions found by a cost-efficient greedy method and by selecting the best singleton item. Recently, the approximation factor of this “best-of-two” algorithm was shown to be within [1/0.462, 1/0.427] (Feldman et al. 2020). A better 2-approximation is achieved by another greedy variant that returns the best solution among the solutions found by a cost-efficient greedy algorithm and all its intermediate solutions, each augmented with the best single additional item (Yaroslavtsev et al. 2020).

### Submodularity for a sequence function

A sequential utility function is defined as $f: {\mathcal {S}} \rightarrow {\mathbb {R}}$, where ${\mathcal {S}}$ is the set of all possible sequences of subsets of a ground set of items $V$. Note that a set function can be seen as a special sequence function, in which the diminishing-returns effect holds for any subsequence relation. Streeter and Golovin (2008) and Zhang et al. (2012) introduce a notion of *string submodularity*, which restricts the diminishing returns to only the prefix subsequence relation. That is to say, a function *f* is string submodular if appending an item to a sequence results in no larger marginal gain than appending the item to a prefix of the sequence. The goal is to find a sequence of a given length that maximizes the value of the function *f*. In our formulation, the sum of multiple submodular functions remains submodular, and thus, string submodular. However, the analysis in the prior work does not apply in our case as we assume that each submodular function is associated with a different budget constraint.

### Submodular ranking

Azar and Gamzu (2011) propose the *submodular ranking* ($\text {SR}$) problem, which aims to find a permutation to minimize the average “cover time” of a set of submodular functions, where we say that an input sequence “covers” a function if it evaluates to the maximum value of the function, and the “cover time” of a sequence of items is the shortest prefix of the sequence for which the function is covered. The problem we study in this paper can be seen as a dual problem of the $\text {SR}$ problem. The $\text {SR}$ problem originates from the classic min-sum set cover ($\text {MSSC}$) problem (Feige et al. 2004) and its generalizations (Azar et al. 2009; Gamzu 2010).

### Diversified web search

In web search, in the absence of the explicit user intent, it is desirable to provide a sequence of high-quality and diverse documents that account for the interests of the overall user population. Typically, the diversity is evaluated by the coverage at the topical level of some existing taxonomy (Zhai et al. 2015). Carbonell and Goldstein (1998) propose a greedy algorithm with respect to *maximal marginal relevance* (MMR) to reduce the redundancy among returned documents. Bansal et al. (2010) define the problem of finding an ordering of search results that maximizes the discounted cumulative gain (DCG), i.e., the sum of discounted gains of different user types, where the discount factor increases if a user type is satisfied later on. They show that, in some special cases, the DCG metric can be rewritten as a weighted sum of submodular functions. Our framework contributes to this theme by, for example, casting each user type or topic as a submodular function.

## Problem definition

We are given a universe set $$V$$ with $$|V |=n $$ items, a set of $$m$$ non-decreasing submodular functions $$F =\{ f _1, \ldots , f _m \}$$, and a cost function $$c: V \rightarrow {\mathbb {R}} _+$$. Recall that a set function $$f: 2^V \rightarrow {\mathbb {R}} _+$$ is non-decreasing if $$f (T) \le f (S)$$ for every $$T \subseteq S \subseteq V $$, and it is submodular if $$f (T \cup \{v \}) - f (T)\ge f (S \cup \{v \}) - f (S)$$ for every $$T \subseteq S \subseteq V $$ and $$v \in V \setminus S $$. Furthermore, each function $$f _i$$ is associated with a budget $$b _i \in {\mathbb {R}} _+$$. We will often write $$f (v \mid S)$$ to mean $$f(\{v\} \cup S) - f(S)$$.

Let $\sigma (V)$ denote the set of permutations of $V$, that is, $\sigma (V) =\{ \pi : V \rightarrow V \mid \pi \text { is a permutation}\}$. Our goal is to find a permutation $\pi \in \sigma (V)$ to maximize the sum of function values $f _i(\pi _{\ell _i})$, where the input set $\pi _{\ell _i}$ is a prefix of the sought permutation $\pi$ with feasibility constraints. In particular, we consider that each function $f _i$ receives as input the *maximal prefix* of $\pi$ that fits within its corresponding budget $b _i$. In other words, the permutation $\pi$ can be seen as a sequence of nested sets, one for each function. Formally, the *max-submodular ranking* ($\text {MSR}$) problem that we study in this paper is defined as follows.

### Problem 1

(Max-submodular ranking ($\text {MSR}$)) Given a set of items $V$, a set of non-decreasing and submodular functions $F =\{ f _1, \ldots , f _m \}$, a cost function $c: V \rightarrow {\mathbb {R}} _+$, and non-negative budgets $b _i$ for each function $f _i$, the $\text {MSR}$ problem aims to find a permutation $\pi \in \sigma (V)$ that maximizes the sum

1 $$\begin{aligned}&\sum _{f _i \in F} f _i(\pi _{\ell _i}),\text {such that }~ \ell _i = \max \{ j \in [n ] : c (\pi _j) \le b _i \}, \end{aligned}$$

where $\pi _j$ is the prefix of the permutation $\pi$ of length *j* and $c (\pi _j) = \sum _{v \in \pi _j} c (v)$.

We make a number of observations for Problem 1.

Without loss of generality, we can assume that $f _i(\emptyset )=0$; otherwise we can translate the objective function by $\sum _{f _i \in F} f _i(\emptyset )$.

Also note that not all items in the permutation solution $\pi$ will necessarily be used as an input to some function $f _i\in F$. Instead, only the items in $\pi _{\ell _i}$ for the largest $\ell _i$ will be used. For this reason, we can think that the output to the $\text {MSR}$ problem is a *partial* permutation; after all functions deplete their budget, the remaining items of the permutation does not matter.

Finally, note that when the cost function $c$ is uniform, i.e., $c (\cdot )=1$, we can consider only integral budget $b _i$ and assume $\ell _i = b _i$.

With respect to the hardness of approximation of the $$\text {MSR}$$ problem, we observe that $$\text {MSR}$$ is equivalent to the standard submodularity-maximization problem when $$m =1$$, that is, when there is only one function in $$F$$. A second reduction from the standard submodularity-maximization problem can be obtained by letting $$b _i=b $$, for all $$i=1,\ldots ,m $$, i.e., when the same budget is used for all functions. The reason is that in this case the sum of submodular functions remains submodular, and we ask to maximize a submodular function under a cardinality constraint. We conclude the following hardness result.

### Remark 1

(Nemhauser and Wolsey 1978). For solving the max-submodular ranking ($\text {MSR}$) problem, no algorithm requiring a polynomial number of function evaluations can achieve a better approximation guarantee than $e/(e-1)$.

It is also well-known that *maximum* *k-cover*, a special case of submodular maximization, is a dual problem to the *minimum set cover* problem, where the constraint in one problem is treated as the objective function in the other (Feige 1998). More generally, the $\text {MSR}$ problem can be considered as the dual problem to the *submodular-ranking* problem ($\text {SR}$) (Azar and Gamzu 2011), whose goal is to find a (partial) ranking of the items so as to minimize the average “cover time” of all functions.

We conclude the section by introducing some additional notation that will be used in our analysis. The optimal permutation is denoted by $\pi ^{*}$. We use the operator $\oplus$ to denote sequence concatenation and overload operator $\subseteq$ for subsequence relation.

## Cardinality constraints

We start our analysis of the $\text {MSR}$ problem for the case of cardinality constraints, that is, when the item costs are uniform ($c (\cdot )=1$). For this particular case we present two algorithms, called $\text {Greedy-U}$ and $\text {Greedy-W}$, both having provable guarantees. Both algorithms generate a permutation by greedily selecting one item before the next. Pseudocode for both algorithms is shown in a unified manner in Algorithm 1. The difference in the two algorithms lies in adopting different coefficients $\alpha _i$, associated with the submodular functions $f _i$, in their selection criteria. The first algorithm, $\text {Greedy-U}$, is an unweighted greedy ($\alpha _i=1$) with respect to the submodular functions $f _i$. The second algorithm, $\text {Greedy-W}$, is a weighted greedy ($\alpha _i=1/b _i$) that puts more weight on functions with smaller budget.

The worst-case running time of both algorithms is $${\mathcal {O}} (n ^2 m)$$. In practice, they run much faster and their actual running time grows almost linearly in $$n $$, thanks to applying a standard lazy evaluation technique (Leskovec et al. 2007). More details on scalability are discussed in Sect. 6.4.
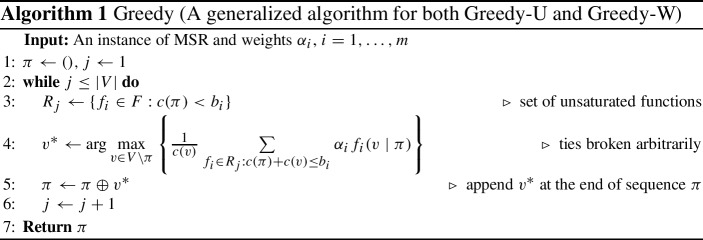


### Unweighted greedy

We show that the unweighted greedy algorithm ($\alpha _i=1$) achieves a 2-approximation guarantee for the $\text {MSR}$ problem with uniform cost. In addition, we show that the approximation ratio is tight for this particular algorithm.

#### Theorem 1

$\text {Greedy-U}$ (Algorithm 1 with coefficients $\alpha _{i}=1$) is a 2-approximation algorithm for the $\text {MSR}$ problem with uniform item costs $(c (\cdot )=1)$.

#### Proof

Write $R _j=\{ f _i \in F: c (\pi _{j-1}) < b _i \}$. By the greedy selection criteria, we get that for arbitrary item $v \in V$ in the *j*-th iteration it holds that

2 $$\begin{aligned} \sum _{f _i \in R _{j}} \left( f _i(\pi _{j}) - f _i(\pi _{j-1}) \right) \ge \sum _{f _i \in R _{j}} f _i(v \mid \pi _{j-1}). \end{aligned}$$

The main idea of the proof is to choose an appropriate item *v* for the above inequality at each iteration of the greedy, and sum over all iterations. We denote the *j*-th item of the optimal permutation $\pi ^{*}$ by $v^{*} _j$. We write $\text {ALG}$ to denote the value achieved by the $\text {Greedy-U}$ algorithm. Then

$$\begin{aligned} \text {ALG}&= \sum _{f _i \in F} f _i(\pi _{b _i}) \\&= \sum _{f _i \in {F}} \sum _{j=1}^{b _i} \left( f _i(\pi _{j}) - f _i(\pi _{j-1}) \right)&\triangleright \text {telescoping series} \\&= \sum _{j=1}^{n} \sum _{f _i \in R _{j}} \left( f _i(\pi _{j}) - f _i(\pi _{j-1}) \right)&\\&\ge \sum _{j=1}^{n} \sum _{f _i \in R _{j}} f _i(v^{*} _{j} \mid \pi _{j-1})&\triangleright \text {Equation}~(2) \\&= \sum _{f _i \in {F}} \sum _{j=1}^{b _i} f _i(v^{*} _{j} \mid \pi _{j-1}) \\&\ge \sum _{f _i \in F} \sum _{j=1}^{b _i} f _i(v^{*} _{j} \mid \pi _{b _i})&\triangleright \text {submodularity} \\&\ge \sum _{f _i \in F} \left( f _i(\pi ^{*} _{b _i} \cup \pi _{b _i}) - f _i(\pi _{b _i}) \right)&\triangleright \text {submodularity} \\&\ge \sum _{f _i \in F} \left( f _i(\pi ^{*} _{b _i}) - f _i(\pi _{b _i}) \right)&\triangleright \text {monotonicity} \\&= \text {OPT}- \text {ALG}. \end{aligned}$$

Consequently, $2\text {ALG} \ge \text {OPT}$, proving the claim. $\square$

We complete the analysis of the $\text {Greedy-U}$ algorithm for the $\text {MSR}$ variant with cardinality constraints, by showing that the approximation ratio 2 is tight.

#### Remark 2

$\text {Greedy-U}$ (Algorithm 1 with coefficients $\alpha _{i}=1$) cannot do better than 2-approximation for the $\text {MSR}$ problem with uniform item costs $(c (\cdot )=1)$.

#### Proof

We construct an instance where the algorithm returns $$\text {ALG} = \frac{1}{2} \text {OPT} $$. The main idea is to force the algorithm to pick up items that are only beneficial to functions with large budget and “starve” those with small budget in the early iterations. Consider functions $$f _i$$ with budget $$b _i=i$$, for all $$i \in [m ]$$. Let $$m =n $$ be even, that is $$m =n = 2k$$ for some *k*. Select $$\epsilon > 0$$. For $$i \le k$$, we define $$f _i(\pi ) = \min \{1, I[v_i \in \pi ] + \epsilon I[v_{i + k} \in \pi ]\}$$, where $$I[\cdot ]$$ is the indicator function. For $$i > k$$, we define $$f _i(\pi ) = I[v_i \in \pi ]$$.

Clearly every $f _i$ is non-decreasing and submodular. One possible optimal permutation is $\pi ^{*} =(v_1,\ldots ,v_n)$, which leads to $\text {OPT} =m$. Algorithm 1 with coefficient $\alpha _i=1$ returns a permutation (out of many equivalent possible permutations) $\pi =(v_n,\ldots ,v_1)$ with $\text {ALG} =(1+\epsilon ) m/2$. By letting $\epsilon$ be arbitrarily small, we see that the bound in Theorem 1 is tight. $\square$

### Weighted greedy

Inspired by the instance that yields the tight bound in Remark 2, it is reasonable to let the algorithm favor functions with small budget at the early iterations. Such a strategy is desirable as it in some sense suggests fairness in resource allocation, i.e., more functions can afford at least one item from the returned ranking. It also turns out to have better performance in experiments. We show that such a strategy is indeed reliable by proving a constant-factor approximation guarantee.

#### Theorem 2

$\text {Greedy-W}$ (Algorithm 1 with coefficients $\alpha _{i}=1/b _i$) is a 3-approximation algorithm for the $\text {MSR}$ problem with uniform item costs $(c (\cdot )=1)$.

#### Proof

Write $R _j=\{ f _i \in F: c (\pi _{j-1}) < b _i \}$. By the greedy selection criteria, we know that for an arbitrary item $v \in V$ it holds that

3 $$\begin{aligned} \sum _{f _i \in R _{j}} \alpha _i (f _i(\pi _{j}) - f _i(\pi _{j-1})) \ge \sum _{f _i \in R _{j}} \alpha _i f _i(v \mid \pi _{j-1}). \end{aligned}$$

We denote by $v^{*} _j$ the *j*-th item of the optimal permutation $\pi ^{*}$. The idea is to replace the arbitrary item *v* with $v^{*} _k \in \pi ^{*}$ and compute a weighted sum. In order to define the weights, given $k < j$, we write $d _{jk} = 1/2$, and $d _{jj} = (j + 1)/2$. Immediately, $\sum _{k \in [j]} d _{jk} = (j - 1)/2 + (j + 1)/2 = j$.

Now Equation (3) implies

$$\begin{aligned} \sum _{f _i \in F} \sum _{j \in [b _i]} j \alpha _i (f _i(\pi _{j}) - f _i(\pi _{j-1}))&= \sum _{j \in [n ]} j \sum _{f _i \in R _{j}} \alpha _i (f _i(\pi _{j}) - f _i(\pi _{j-1})) \\&= \sum _{j \in [n ]} \sum _{k \in [j]} d _{jk} \sum _{f _i \in R _{j}} \alpha _i (f _i(\pi _{j}) - f _i(\pi _{j-1})) \\&\ge \sum _{j \in [n ]} \sum _{k \in [j]} d _{jk} \sum _{f _i \in R _{j}} \alpha _i f _i(v^{*} _{k} \mid \pi _{j-1}). \end{aligned}$$

We will denote the left hand side of the above equation by LHS, and the right hand side by RHS. We will first bound the RHS. In order to do so, we need an additional bound on the weights $d _{jk}$, namely, for any fixed *k*,

4 $$\begin{aligned} \sum _{j = k}^{b} \frac{d _{jk}}{b} = \frac{k + 1}{2b} + \frac{b - k}{2b} = \frac{b + 1}{2b} > \frac{1}{2}. \end{aligned}$$

We can now bound the right hand side with

$$\begin{aligned} \text {RHS}&= \sum _{f _i \in F} \sum _{j \in [b _i]} \sum _{k \in [j]} d _{jk} \alpha _i f _i(v^{*} _{k} \mid \pi _{j-1})\\&= \sum _{f _i \in F} \sum _{k \in [b _i]} \sum _{j = k}^{b _i} d _{jk} \alpha _i f _i(v^{*} _{k} \mid \pi _{j-1})\\&\ge \sum _{f _i \in F} \sum _{k \in [b _i]} \sum _{j = k}^{b _i} d _{jk} \alpha _i f _i(v^{*} _{k} \mid \pi _{b _i})&\triangleright \text {submodularity} \\&\ge \sum _{f _i \in F} \sum _{k \in [b _i]} f _i(v^{*} _{k} \mid \pi _{b _i}) /2&\triangleright \text {Equation}~(4) \\&\ge \sum _{f _i \in F} (f _i(\pi ^{*} _{b _i} \cup \pi _{b _i}) - f _i(\pi _{b _i})) /2&\triangleright \text {submodularity} \\&\ge \sum _{f _i \in F} (f _i(\pi ^{*} _{b _i}) - f _i(\pi _{b _i})) /2&\triangleright \text {monotonicity} \\&= (\text {OPT}- \text {ALG}) /2. \end{aligned}$$

Now we consider the left hand side,

$$\begin{aligned} \text {LHS}&= \sum _{f _i \in F} \sum _{j \in [b _i]} \frac{j}{b _i} (f _i(\pi _{j}) - f _i(\pi _{j-1}))\\&= \sum _{f _i \in F} \left( \frac{b _i}{b _i} f _i(\pi _{b _i}) - \sum _{j < b _i} \frac{j+1-j}{b _i} f _i(\pi _{j}) \right) \\&\le \sum _{f _i \in F} f _i(\pi _{b _i}) \\&= \text {ALG}. \end{aligned}$$

Putting everything together, $\text {ALG} \ge \text {LHS} \ge \text {RHS} \ge (\text {OPT}- \text {ALG}) /2,$ and we obtain $3\text {ALG} \ge \text {OPT}$. $\square$

## Knapsack constraints

The traditional way of handling knapsack constraints is to adopt a cost-efficient variant of the greedy algorithm where in each iteration we select the item with the largest ratio between utility and cost. Furthermore, we compute a second solution by selecting the maximum-utility singleton item that is feasible. The idea is to use the second solution to rescue the situation in which the greedy algorithm starts with some cost-efficient small items and then is “starved” (i.e., the remaining budget is not enough to admit another valuable large item). This idea however falls short when it comes to the $\text {MSR}$ problem. The reason is that there are multiple knapsacks and each one of them may be “starved” by different big items. A more sophisticated way is needed to compute an alternative second solution.

We now discuss our proposed method in more detail. First, an item $v \in V$ is called *large* with respect to a function $f _i \in F$ if its cost is more than half of the budget $b _i$, that is, $2c (v) > b _i$. It is obvious that a function $f _i$ can afford at most one large item. The following variant of the $\text {MSR}$ problem targets a similar objective to that of $\text {MSR}$, but exclusive to only large items.

### Problem 2

(Max-submodular ranking of large items ($\text {MSRL}$)) Given a set of items $V$, a set of non-decreasing and submodular functions $F =\{ f _1, \ldots , f _m \}$, a cost function $c: V \rightarrow {\mathbb {R}} _+$, and non-negative budgets $b _i$ for each function $f _i$, the $\text {MSRL}$ problem aims to find a permutation $\pi \in \sigma (V)$ that maximizes

5 $$\begin{aligned} z (\pi ) = \sum _{v_j \in \pi } z (v_j, c (\pi _{j-1})) = \sum _{v_j \in \pi } \sum _{f _i \in F (v_j;\pi )} f _i(v_j), \end{aligned}$$

where $$F (v_j;\pi )$$ is the set of functions that take the *j*-th item $$v_j \in \pi $$ as a large item, i.e., $$F (v_j;\pi ) = \{ f _i \in F: 2c (v_j) > b _i, c (\pi _{j}) \le b _i \}$$, and $$z (v_j, c)$$ is defined to be the contribution of item $$v_j$$ by appending it to a prefix with cost $$c $$.

We start by proving that the cost-efficient greedy algorithm yields a 3-approximation when there is no large item in $\pi ^{*}$. Next, we devise a dynamic programming ($\text {DP}$) algorithm in Algorithm 2 to approximately solve $\text {MSRL}$. Finally, we prove that the best solution among the greedy solution and the $\text {DP}$ solution can achieve an approximation guarantee that is arbitrarily close to 4.

*Step 1: bounding small items in* $\pi ^{*}$. We first discuss the case in the absence of large items in $\pi ^{*}$. Let us introduce some notation. We denote the *j*-th selected item by our algorithm by $u_j$. We denote the *k*-th item of the optimal permutation $\pi ^{*}$ by $v^{*} _k$. We denote the greedy solution of Algorithm 1 with coefficient $\alpha _{i}=1$ by $\text {ALG} _1$ and the $\text {DP}$ solution of Algorithm 2 by $\text {ALG} _2$.

The next theorem shows that, if every function $f _i$ includes no such large item in $\pi ^{*}$, $\text {ALG} _1$ ensures a constant-factor guarantee. Otherwise, we have an additional term $z (\pi ^{*})$, which we will bound later.

### Theorem 3

The greedy algorithm yields $3\text {ALG} _1 + z (\pi ^{*}) \ge \text {OPT}$.

The proof relies on the next technical observation.

### Observation 1

For any *k*, if item $v^{*} _k \in \pi ^{*}$ is feasible and not large for function $f _i$, i.e., $c (\pi ^{*} _{k}) \le b _i$ and $2c (v^{*} _k) \le b _i$, then at the *j*-th greedy iteration such that $c (\pi _{j-1}) \le c (\pi ^{*} _{k})/2$, we have $c (\pi _{j-1}) + c (v^{*} _k) \le b _i$.

### Proof

The proof is straightforward by combining $c (\pi _{j-1}) \le c (\pi ^{*} _{k})/2 \le b _i/2$ and $c (v^{*} _k) \le b _i/2$. $\square$

### Proof of of Theorem 3

Write $R _j=\{ f _i \in F: c (\pi _{j-1}) < b _i \}$. By greedy, we know that for arbitrary item $v \in V$ in the *j*-th iteration it holds that

6 $$\begin{aligned} \frac{1}{c (u_j)} \sum _{f _i \in R _{j}: c (\pi _{j}) \le b _i} f _i(u_j \mid \pi _{j-1})&\ge \frac{1}{c (v)} \sum _{f _i \in R _{j}: c (\pi _{j-1})+c (v) \le b _i} f _i(v \mid \pi _{j-1}). \end{aligned}$$

To simplify the notation used in the above inequality, let us define $X_j = \{i \in [m ] \mid c (\pi _{j}) \le b _i\}$ to be the valid function indices for $\pi _j$, and similarly $Y_{jk} = \{i \in [m ] \mid c (\pi _{j-1})+c (v^{*} _k) \le b _i\}$.

For function $f _i$, we define $\ell ^* _i = \max \{ j \in [n ] : c (\pi ^{*} _j) \le b _i \}$.

Let us define a sequence of weights $d _j= \text {len} (A_j)$, where the interval $A_j = (c (\pi _{j-1}), c (\pi _{j})] \cap (0, c (\pi ^{*})/2]$.

We will start by lower bounding $\text {ALG} _1$ with

$$\begin{aligned} \text {ALG} _1&= \sum _{f _i \in F} \sum _{j \in [\ell _i]} f _i(u_j \mid \pi _{j-1})= \sum _{j \in [n ]} \sum _{i \in X_j} f _i(u_j \mid \pi _{j-1})&\\&\ge \sum _{j \in [n ]} \frac{d _j}{c (u_j)} \sum _{i \in X_j} f _i(u_j \mid \pi _{j-1}).&\triangleright \text {since }d _j \le c (u_j) \end{aligned}$$

Let us denote the right hand side with *C*. We will prove the theorem by showing that $C \ge (\text {OPT}- \text {ALG} _1 - z (\pi ^{*})) /2$.

We define $d _{jk} = \text {len} (A_j \cap B_k)$, where interval $B_k = (c (\pi ^{*} _{k-1})/2, c (\pi ^{*} _{k})/2]$. We see immediately that $d _{j} = \text {len} (A_j) = \sum _{k \in [n ]}d _{jk}$ as $B_k$ partition $A_j$. Similarly, $\sum _{j \in [n ]} d _{jk} = \text {len} (B_k) = c (v^{*} _k)/2$ as $A_j$ partition $B_k$.

We first claim that for any *i*,

7 $$\begin{aligned} \text {if}\quad j > \ell _i \quad \text {and}\quad k \le \ell ^* _i, \quad \text {then}\quad d_{jk} = 0. \end{aligned}$$

To prove Equation (7) note that $j - 1 \ge \ell _i$ implies that $c (\pi _{j - 1}) \ge b_i$ while $k \le \ell ^* _i$ implies that $c (\pi ^{*} _{k}) \le b_i$. Consequently, $A_j \cap B_k = \emptyset$ and $d_{jk} = 0$.

Let us now define $S_i = \left\{ k \in [\ell ^* _i] : 2c (v^{*} _k) \le b_i \right\}$ to be the set of small items for the *i*-th function. We claim that

8 $$\begin{aligned} \text {if}\quad k \in S_i \quad \text {and}\quad d_{jk} > 0, \quad \text {then}\quad c (\pi _{j-1})+c (v^{*} _k) \le b _i. \end{aligned}$$

To prove Equation (8) note that since $k \le \ell ^* _i$, we have $c (\pi ^{*} _{k}) \le b _i$. Moreover, since $k \in S_i$, we have $2c (v^{*} _k) \le b _i$. If $c (\pi _{j-1}) > c (\pi ^{*} _k) / 2$, then $A_j \cap B_k = \emptyset$ and so $d_{jk} = 0$. Thus, $c (\pi _{j-1}) \le c (\pi ^{*} _k) / 2$. Observation 1 now proves Equation (8).

We can now lower bound *C* with

$$\begin{aligned} C&= \sum _{j \in [n ]} \sum _{k \in [n ]} \frac{d _{jk}}{c (u_j)} \sum _{i \in X_j} f _i(u_j \mid \pi _{j-1})&\triangleright \text {since } d _{j} = \sum _{k \in [n ]}d _{jk} \\&\ge \sum _{j \in [n ]} \sum _{k \in [n ]} \frac{d _{jk}}{c (v^{*} _k)} \sum _{i \in Y_{jk}} f _i(v^{*} _k \mid \pi _{j-1})&\triangleright \text {Equation}~(6) \\&= \sum _{i \in [m ]} \sum _{k \in [n ]} \sum _{j \in [\ell _i]: i \in Y_{jk}} \frac{d _{jk}}{c (v^{*} _k)} f _i(v^{*} _k \mid \pi _{j-1})&\\&\ge \sum _{i \in [m ]} \sum _{k \in S_i} \sum _{j \in [\ell _i]: i \in Y_{jk}, d _{jk} > 0} \frac{d _{jk}}{c (v^{*} _k)} f _i(v^{*} _k \mid \pi _{j-1}) \\&= \sum _{i \in [m ]} \sum _{k \in S_i} \sum _{j \in [\ell _i]} \frac{d _{jk}}{c (v^{*} _k)} f _i(v^{*} _k \mid \pi _{j-1})&\triangleright \text {Equation}~(8) \\&\ge \sum _{i \in [m ]}\sum _{k \in S_i} \sum _{j \in [\ell _i]} \frac{d _{jk}}{c (v^{*} _k)} f _i(v^{*} _{k} \mid \pi _{\ell _i})&\triangleright \text {submodularity} \\&= \sum _{i \in [m ]}\sum _{k \in S_i} \sum _{j \in [n ]} \frac{d _{jk}}{c (v^{*} _k)} f _i(v^{*} _{k} \mid \pi _{\ell _i})&\triangleright \text {Equation}~(7) \\&= \sum _{i \in [m ]}\sum _{k \in S_i}f _i(v^{*} _{k} \mid \pi _{\ell _i}) /2&\triangleright \text {since } \sum _{j \in [n ]} d _{jk} = c (v^{*} _k)/2 \\&\ge -z (\pi ^{*})/2 + \sum _{i \in [m ]}\sum _{k \in [\ell ^* _i]}f _i(v^{*} _{k} \mid \pi _{\ell _i}) /2 \\&\ge -z (\pi ^{*})/2 + \sum _{i \in [m ]} (f _i(\pi ^{*} _{\ell ^* _i} \cup \pi _{\ell _i}) - f _i(\pi _{\ell _i})) /2&\triangleright \text {submodularity} \\&\ge -z (\pi ^{*})/2 + \sum _{i \in [m ]} (f _i(\pi ^{*} _{\ell ^* _i}) - f _i(\pi _{\ell _i})) /2&\triangleright \text {monotonicity} \\&= (\text {OPT}- \text {ALG} _1 - z (\pi ^{*})) /2. \end{aligned}$$

Putting everything together, we obtain $\text {ALG} _1 \ge (\text {OPT}- \text {ALG} _1 - z (\pi ^{*})) /2$, that is, $3\text {ALG} _1 + z (\pi ^{*}) \ge \text {OPT}$. $\square$

*Step 2: bounding large items in* $\pi ^{*}$. When some functions do take large items in $\text {OPT}$, the quantity $z (\pi ^{*})$ is positive, and we need to bound it. We will do this by solving approximately the $\text {MSRL}$ problem.

Our first result allows to order items based on their cost when solving $\text {MSRL}$.

### Theorem 4

Assume a permutation $\pi$ with some item $v_i$ for which there is an index $j < i$ such that $c (v_j) \ge c (v_i)$. Define a sub-permutation $\pi '$ by removing $v_i$. Then $z (\pi ') \ge z (\pi )$.

The proof relies on the following technical observation.

### Observation 2

Given an item *v* and two sequences $\pi ,\pi '$ with costs $c (\pi ) \le c (\pi ')$, we have $F (v;\pi ' \oplus v) \subseteq F (v;\pi \oplus v)$ and $z (v; c (\pi )) \ge z (v; c (\pi '))$.

### Proof

Note that

$$\begin{aligned} F (v;\pi \oplus v)&= \{ f _i \in F: 2c (v)> b _i, c (\pi )+ c (v) \le b _i \} \\&\supseteq \{ f _i \in F: 2c (v) > b _i, c (\pi ')+ c (v) \le b _i \} = F (v;\pi ' \oplus v). \end{aligned}$$

Consequently, we have

$$\begin{aligned} z (v; c (\pi )) = \sum _{f _i \in F (v;\pi \oplus v)} f _i(v) \ge \sum _{f _i \in F (v;\pi ' \oplus v)} f _i(v) = z (v; c (\pi ')), \end{aligned}$$

proving the claim. $\square$

### Proof of Theorem 4

Let $$v_i$$ be an item that is in $$\pi $$ but not in $$\pi '$$. Assume that $$2c (v_i) > b $$ for arbitrary function budget $$b $$. Then $$c (\pi _{i - 1}) + c (v_i) \ge 2 c (v_i) > b $$, following the assumptions of the theorem. Consequently, $$F (v;\pi _i) = \emptyset $$ and $$z (v_i, c (\pi _{i - 1})) = 0$$. Let $$u_j$$ be the *j*-th item in $$\pi '$$. Observation 2 now implies that

$$\begin{aligned} z (\pi ) = \sum _{v_i \in \pi } z (v_i; c (\pi _{i-1})) = \sum _{v_i \in \pi '} z (v_i; c (\pi _{i-1})) \le \sum _{u_j \in \pi '} z (u_j; c (\pi _{j-1}')) = z (\pi '), \end{aligned}$$

proving the claim. $\square$

The above theorem enables a way to limit ourselves to sequences of large items with non-decreasing costs when solving $\text {MSRL}$.

Let us assume for simplicity that $z (\cdot )$ is an integer-value in [*k*]. We will discuss how to relax this assumption shortly.

We can solve $\text {MSRL}$ by constructing a table $T$ with entry $T (a, j)$ for each value $a \in [k]$ and each item with index $j \in [n ]$. We define the entry $T (a, j)$ to be the lowest possible cost of a permutation using only the first *j* items with at least value $a$,

$$\begin{aligned} T (a, j) = \min \{c (\pi ) \mid z (\pi ) \ge a, \pi \subseteq (v_1, \ldots , v_j) \}. \end{aligned}$$

Note that it is also possible to solve $\text {MSRL}$ by defining a different dual $\text {DP}$, where each entry $T (b, j)$ contains the highest value realizable by a permutation using only the first *j* items with at most cost $b$. However, this dual $\text {DP}$ is not amenable to the standard rounding trick we will introduce shortly.

### Theorem 5

The table $$T $$ satisfies the following relation:

9 $$\begin{aligned} T (a, j) = \min \left\{ T (a, j-1), \min _{a ' \mid a ' + z ({v_j}; T (a ', j - 1)) \ge a} T (a ', j - 1) + c (v_j)\right\} , \end{aligned}$$

when $j > 1$. Moreover, $T (0, 1) = 0$, $T (a, 1) = c (v_1)$ if $0 < a \le z (v_1)$, and $\infty$ otherwise.

### Proof

We will prove by induction. The result holds trivially for $T (a,1)$.

Next, we assume the theorem holds for all $T (a ', j - 1)$. Now we examine $T (a,j)$. Let $\pi$ be a sequence responsible for $T (a, j)$. Let *X* be the value of the right hand side of Equation 9. Clearly, we have $X \ge c (\pi )$, and we now prove the claim by showing that $X \le c (\pi )$.

If $v_j$ not in $\pi$, then $X \le T (a, j - 1) \le c (\pi )$, and we are done. If $v_j$ is in $\pi$, then let $\pi '$ be the permutation without $v_j$. Let $a ' = z (\pi ')$, and by the inductive hypothesis, we know that $T(a ', j - 1) \le c (\pi ')$. Then

$$\begin{aligned} a \le z (\pi ) = a ' + z (v_j; c (\pi ')) \le a ' + z (v_j; T(a ', j - 1)), \end{aligned}$$

where the last inequality is by Observation 2. Therefore, according to the $\text {DP}$ updating rule, we have

$$\begin{aligned} X \le T(a ', j - 1) + c (v_j) \le c (\pi ') + c (v_j) = c (\pi ), \end{aligned}$$

completing the proof. $\square$

We can use Theorem 5 to construct $$T $$ using a dynamic program, which is described in Algorithm 2. Next, we will show that the $$\text {DP}$$ solves the $$\text {MSRL}$$ problem. 
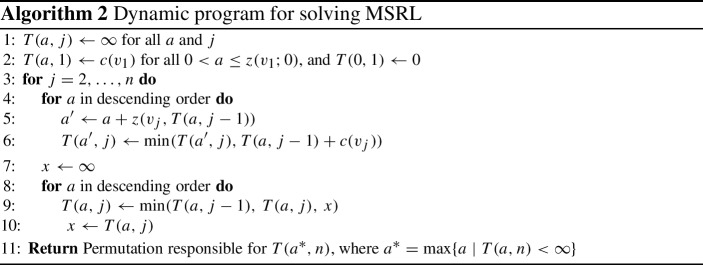


### Theorem 6

Assume that $z (\pi )$ is an integer in [*k*] for every $\pi$. The permutation $\pi$ responsible for $T (a ^*, n)$, where $a ^* = \max \{a \mid T (a, n) < \infty \}$, returned by Algorithm 2 has the largest $z (\cdot )$ value. Besides, Algorithm 2 runs in ${\mathcal {O}} (n(k + m) + m \log m)$ time.

### Proof

The correctness of the algorithm follows directly from Theorem 5. There are in total $$k \times n $$ table entries. Note that we can avoid directly invoking $$z (v_j; \cdot )$$, which alone needs time $${\mathcal {O}} (m)$$, by sorting $$f _i$$ by their budget $$b _i$$ and gradually including more $$f _i$$ as $$c (T (a, j - 1))$$ and $$a $$ decrease. This leads to an additional $${\mathcal {O}} (m)$$ time per index *j*. $$\square $$

We provide a numerical example to illustrate the $\text {DP}$ algorithm.

### Example 1

Consider two modular functions $f _1,f _2$ with budget $b _1=3,b _2=9$, and three items $v_1,v_2,v_3$ with costs 2.5, 3, 6.5, respectively. We define $f _1(v_1) = 1$, $f _1(v_2) = 1.5$, $f _2(v_3) = 1$, and 0 otherwise.

It is easy to see that both the cost-efficient greedy algorithm and the best singleton will pick item $v_2$, which leads to a sub-optimal ranking, while the $\text {DP}$ algorithm can help us find the optimal ranking.

The $$\text {DP}$$ algorithm first initializes $$T (a, j) \leftarrow \infty $$ for all $$a $$ and *j*. We then process items $$v_1,v_2,v_3$$ in non-decreasing order by their costs.

- Item $v_1$: we set $T (a, 1) = c (v_1)$ for all $0 < a \le f _1(v_1)$ and $T (0, 1) = 0$.
- Item $v_2$: we set $T (a, 2) = T (a, 1)$ for all $a \le f _1(v_1)$, and $T (a, 2) = c (v_2)$ for all $f _1(v_1) < a \le f _1(v_2)$.
- Item $v_3$: we set $T (a, 3) = T (a, 2)$ for all $a \le f _1(v_2)$, and $T (a, 3) = c (v_1) + c (v_3)$ for all $f _1(v_2) < a \le f _1(v_1)+f _2(v_3)$.

Finally, we return the permutation $\pi = (v_1,v_3)$ responsible for $T (a ^*, 3)$, where $a ^* = f _1(v_1)+f _2(v_3)$.

So far we have assumed that $$z $$ is an integer. Next, we show that with a standard rounding technique, the $$\text {DP}$$ method in Algorithm 2 gives an $$\text {FPTAS}$$ for $$\text {MSRL}$$. The idea is to apply the $$\text {DP}$$ to a rounded instance, which is obtained by first scaling and rounding down every function $$\lfloor f _i / K \rfloor $$ for certain *K*.

### Theorem 7

Let $P = \max _{i, v} f _i(v)$, where *v* is a large item for $f _i$. Let $K = \frac{P\epsilon }{m}$ for any constant $\epsilon > 0$. Define $f '_i = \lfloor f _i / K \rfloor$ and let $z '(\pi )$ be the score of a permutation using $f '_i$ instead of $f _i$. Let $\pi$ be the permutation with the largest $z (\pi )$. Then $Kz '(\pi ) \ge (1 - \epsilon )z (\pi )$.

### Proof

Due to scaling and rounding down we have $$f _i(v) - K f '_i(v) \le K$$. Since there can be at most one large item per function, and the score $$z $$ contains at most $$m $$ functions, thus, $$z (\pi ) - Kz '(\pi ) \le m K = P \epsilon \le \epsilon z (\pi )$$. $$\square $$

### Corollary 1

Algorithm 2 with rounding yields $1/(1 - \epsilon )$ approximation guarantee in ${\mathcal {O}} (n m ^2 / \epsilon )$ time.

### Proof

Let $$\pi $$ be the permutation with the largest $$z $$ and let $$\pi '$$ be the permutation with the largest $$z '$$. Then $$z (\pi ') \ge Kz '(\pi ') \ge Kz '(\pi ) \ge (1 - \epsilon )z (\pi )$$, proving the approximation guarantee.

To prove the running time note that $z (\cdot ) \le m P$ and $z '(\cdot ) \le m P / K = m^2 / \epsilon$. Theorem 6 proves the claim. $\square$

We are finally ready to state our main result for $\text {MSR}$ with non-uniform cost.

### Theorem 8

The best among Algorithm 1 with coefficient $\alpha _{i}=1$ and Algorithm 2 is $(3+1/(1-\epsilon ))$-approximation for the $\text {MSR}$ problem with non-uniform cost.

### Proof

Theorem 3 and Corollary 1 imply that

$$\begin{aligned} (3 + (1 - \epsilon )^{-1})\text {ALG} \ge 3 \text {ALG} _1 + (1 - \epsilon )^{-1}\text {ALG} _2 \ge \text {ALG} _1 + z (\pi ^{*}) \ge \text {OPT}, \end{aligned}$$

where $\text {ALG} = \max \{ \text {ALG} _1, \text {ALG} _2 \}$, proving the claim. $\square$

## Experimental evaluation

In this section, we evaluate the performance of the proposed algorithms on real-world datasets. We first discuss our experimental evaluation for a playlist-making use-case. We model this use-case using the *max-activation ranking* ($\text {MAR}$) problem, which is a special case of the $\text {MSR}$ problem when the submodular functions $f _i$ are 0–1 functions. We then conduct two experiments for the $\text {MSR}$ problem: (*i*) multiple intents re-ranking and (*ii*) sequential active learning. Finally, we evaluate the running time of our methods. Statistics of the datasets used in the experiments are summarized in Table 1. Our implementation and pre-processing scripts can be found in a Github repository.^1^

### Proposed methods and baselines

The proposed greedy algorithms are denoted by $\text {Greedy-U}$ and $\text {Greedy-W}$; as discussed in Sect. 4. The proposed dynamic program is denoted by $\text {DP}$. As baselines we use the following algorithms.

- The greedy algorithm for the $\text {SR}$ problem (Azar and Gamzu 2011), which favors functions near completion. We refer to this baseline as $\text {AG}$.
- When only the minimum budget among all functions is considered, the objective is a submodular function as a whole. We then consider the well-known “best-of-two” algorithm that returns the best solution among the solutions found by a cost-efficient greedy method and by selecting the best singleton item. We refer to this baseline as $\text {Subm}$.
- A simple ranking method ($\text {Quality}$) that orders individual items in non-increasing quality.
- A random ranking algorithm ($\text {Random}$).

Note that in general, computing the optimal solution requires enumerating all sequences of length equal to the maximum budget, which is computationally intractable even for a modest scenario with universe set $|V |=100$ and budget $b =10$.

**Table 1:** Datasets statistics

Dataset	$n = |V |$	$m = |F |$
Songs	1872	100
Movies	3669	100
Books	3753	1000
20 Newsgroups	172	5
Handwritten Digits	1347	3

### Experiments with the max-activation ranking ($\text {MAR}$) problem

**Fig. 1:**
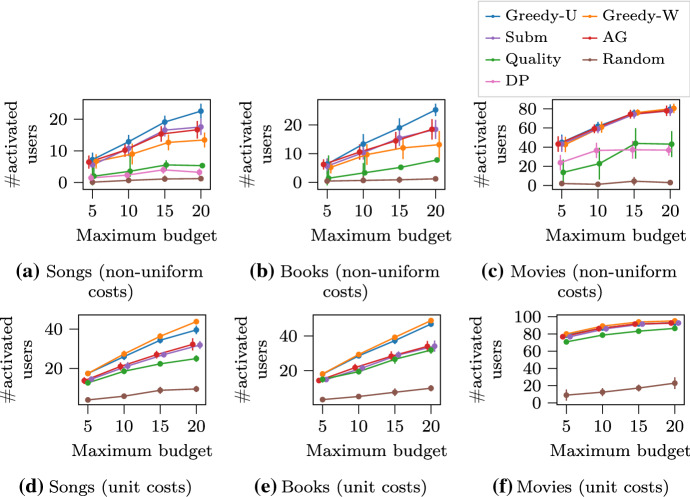
Results of using the $\text {MAR}$ problem formulation for making a playlist of items. The goal is to maximize the number of activated users. The universe $V$ includes songs, movies or books. A user (a 0–1 activation function $f _i$) is activated if they like at least one item among all items they consume within their budget. Markers are jittered horizontally to avoid overlap

We evaluate our methods on three datasets, the Million Song dataset (Bertin-Mahieux et al. 2011), the MovieLens dataset (Harper and Konstan 2015), and the Amazon Review dataset on books category (Ni et al. 2019). The three datasets have similar format, where each record can be seen as a triple of user, item and rating. We describe our experimental evaluation for the first dataset, and the other two datasets are processed in the same way and give very similar results, as can be verified in Fig. 1.

In the Million Song dataset, each record is a triple representing a user, song and play count. We assume that a user likes a song if they play the song more than once. We investigate an instance of the $\text {MAR}$ problem for the application scenario of creating a *playlist*. In particular, we want to find a ranking of songs that maximizes the number of users who like at least one song among songs they listen to. In this case, each user is modeled as a 0–1 activation function. We generate a random budget for each user, i.e., the maximum number of songs a user will listen to, from 1 to a given maximum budget. We also generate a random cost from 1 to 10 for each song in order to experiment with an additional non-uniform cost scenario.

The results of our evaluation are shown in Fig. 1. The error bars are over random user budgets and item costs. In the unit-cost scenario, the proposed $\text {Greedy-W}$ algorithm is the best performing, closely followed by the proposed $\text {Greedy-U}$ algorithm. The performance of the baselines is inferior, and one reason is that they fail to take into account the user budget. In the non-uniform cost scenario, the proposed $\text {Greedy-U}$ algorithm obtains the best performance. Note that it is expected that $\text {DP}$ has poor performance, as it is meant to help in extreme cases. Also note that $\text {DP}$ does not scale for the book-list dataset—more details on scalability are discussed in Sect. 6.4. Interestingly, $\text {Greedy-W}$ performs worse than $\text {AG}$, which indicates that a more sophisticated weighting scheme is needed to combine non-uniform budget and cost.

### Experiments with the max-submodular ranking ($\text {MSR}$) problem

#### Multiple intents re-ranking

We simulate a web-page ranking application for documents in the 20 Newsgroups dataset (Dua and Graff 2017). For each newsgroup, we treat its title as a query, and collect documents that contains the query. We extract 5 topics from the collected documents by means of LDA model (Blei et al. 2003). Subsequently, each topic (i.e., its top 20 keywords) is considered as a potential user intent, and the submodular utility for a particular topic when given a set of documents is the coverage rate of its top keywords. We aim to find a ranking of documents that maximize the total utility of all user intents. As in the previous experiment, we generate a random budget for each user intent, i.e., the maximum number of documents the potential user will read, from 1 to a given maximum budget. For an additional non-uniform cost scenario, we use the document length as the cost for reading a document, and accordingly multiply the budget by the average document length.

**Fig. 2:**
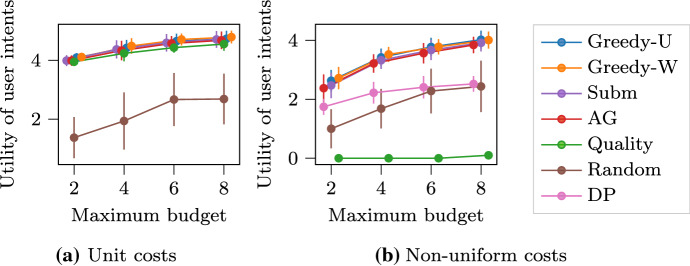
$\text {MSR}$ for multiple intents re-ranking in web page ranking. The goal is to maximize the total utility of all user intents within their individual reading budget. The universe $V$ includes documents. The utility of a user intent (a coverage function $f _i$) is represented by the coverage rate of its top keywords. Markers are jittered horizontally to avoid overlap

The results of our experiment are shown in Fig. 2, where we report the average performance across all newsgroups. In the unit-cost scenario, the top-contender algorithms have close performance. This is due to the overwhelming advantage of lengthy documents that contain more words and produce higher utility. In the more realistic non-uniform cost scenario, our algorithms, $\text {Greedy-U}$ and $\text {Greedy-W}$, achieve the best performance. $\text {Quality}$ algorithm behaves the worst as it fails to consider the cost of items, and its first-rank lengthy document exceeds the user budget most of the time.

#### Sequential active learning

Active learning seeks to make label queries on only a small number of informative data points in order to maximize model performance. In particular, for the *k*-nearest neighbors (*k*NN) model, an intuitive measure for informativeness of a set of labeled data points is the average distance from an unlabeled data point to its closest labeled point, i.e., the facility-location function (Wei et al. 2015). We refer to this average distance as the *radius*. Thus, the active-learning task can be naturally formulated as labeling a small subset of data to maximize the radius reduction. Note that the reduction of the radius by labeling a subset of data points is clearly non-decreasing and submodular.

In our setting, we assume that we have access to multiple models that are trained on the same labeled data, and we aim to label data sequentially to maximize the total reduction in the radii among all models. This happens, for example, when each model runs on a different subset of features. Interestingly, in this case each model can be seen as a student with different learning capacity, and a teacher tries to optimize the classroom teaching by feeding them labeled data (Zhu et al. 2017). We evaluate the performance of active-learning *k*NNs ($k=1$) with Euclidean distance in the Handwritten Digits dataset (Dua and Graff 2017). Each *k*NN model adopts a different strategy in unsupervised feature selection, such as variance thresholding, PCA, and feature agglomeration. Again, we generate a random query budget for each model and a random cost (from 1 to 10) for labeling each data point.

**Fig. 3:**
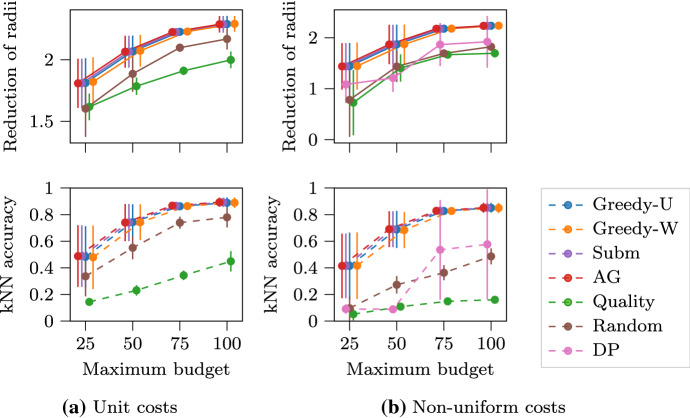
$\text {MSR}$ for sequential data subset selection for *k*NN models. The goal is to boost the average predictive accuracy of *k*NN models. The universe $V$ includes all data points. The sum of the surrogate objective function $f _i$ (reduction of radii) for each model is optimized. Markers are jittered horizontally to avoid overlap

As we can see in Fig. 3, all greedy algorithms are very effective in reducing the radii. The correlation between the radius reduction and model accuracy (over testing data) is obvious. Note that the $\text {Random}$ algorithm is a standard strong baseline in data subset selection, which is outperformed by the greedy algorithms by a large margin. The comparison becomes more evident in the non-uniform cost scenario, as the $\text {Random}$ algorithm fails to take into account the item costs.

### Running time

**Fig. 4:**
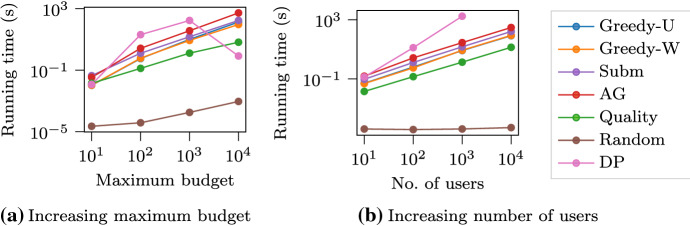
Running time of all methods for the task of making a synthetic playlist

We examine the scalability of all methods by fixing either the number of users (i.e., functions) or the maximum budget (equal to the number of items), while varying the other. In Fig. 4 we demonstrate the running time of all algorithms for the task of making a synthetic playlist. In this case, we generate a dataset by assuming that each user likes a small random subset of items. We generate a random budget for each user, from 1 to the given maximum budget, and a random cost from 1 to 10 for each item.

When comparing the running time, the $\text {Quality}$ algorithm is a meaningful baseline, as it produces a ranking after a single evaluation on each item over all functions, i.e., ${\mathcal {O}} (\max \{n \log (n), m n \})$. Its running time varies almost linearly as a function of the budget, which is in contrast to the behavior of the naïve greedy algorithms. Thanks to the lazy evaluation technique (Leskovec et al. 2007), the running time of all greedy algorithms actually grows nearly linearly in the budget. The $\text {AG}$ algorithm is slower as it is subject to frequent function evaluations, because its greedy criterion depends on the current function values. The running time of the $\text {DP}$ algorithm grows quadratically in the number of functions, which has difficulty in scaling to a very large number. On the other hand, it scales well in the number of items, and particularly, when the budget is big, it finishes quickly as there is no large item. The running time of all except for the $\text {Random}$ algorithm grows linearly in the number of functions, which is inevitable if the utility of items is considered.

## Conclusions

In this paper, we introduce a novel problem in the active area of submodular optimization. Our problem, max-submodular ranking ($\text {MSR}$), ask to find a ranking of items such that the sum of multiple budgeted submodular utility is maximized. The $\text {MSR}$ problem has wide application in the ranking of web pages, ads, and other types of items. We propose several practical algorithms with approximation guarantees for the $\text {MSR}$ problem, with either cardinality or knapsack budget constraints. We empirically demonstrate the superior performance of the proposed algorithms on real-life datasets, compared with a state-of-the-art baseline and other meaningful heuristics.

One direction for future work is to narrow the gap between the approximation ratio and the lower bound. Another direction is to study the online version of the $\text {MSR}$ problem, to allow for the arrival of new submodular functions. Other potential directions include imposing a more general constraint for each submodular function and experimenting with new applications.

## Footnotes

1 https://github.com/Guangyi-Zhang/max-submodular-ranking
